# Berberine Potentiates Insulin Secretion and Prevents β-cell Dysfunction Through the miR-204/SIRT1 Signaling Pathway

**DOI:** 10.3389/fphar.2021.720866

**Published:** 2021-09-22

**Authors:** Xiaoyan Lv, Yali Zhao, Xuehan Yang, Hao Han, Yue Ge, Meishuang Zhang, Hansi Zhang, Ming Zhang, Li Chen

**Affiliations:** ^1^ Department of Pharmacology, College of Basic Medical Sciences, Jilin University, Changchun, China; ^2^ Department of Clinical Laboratory, The Second Hospital of Jilin University, Changchun, China; ^3^ Department of Pharmacology, School of Nursing, Jilin University, Changchun, China

**Keywords:** berberine, type 2 diabetes, β-cell dysfunction, apoptosis, miR-204, SIRT1

## Abstract

Pancreatic β-cell dysfunction is a key link during the progression of type 2 diabetes (T2DM), and SIRT1 participates in the regulation of various physiological activities of islet β-cells. However, as a key link in signal transduction, it is not clear how SIRT1 is regulated. By TargetScan prediction, we found that miR-204, which is enriched in islets, has highly complementary binding sites with SIRT1. Therefore, we speculate that miR-204 may be the upstream regulatory target of SIRT1 in islets and thus participate in the occurrence of β-cell dysfunction. In this study, we explored the association between miR-204 and β-cell dysfunction, the therapeutic effects of berberine (BBR) on β-cell function and the possible mechanisms. We found that miR-204 increased and SIRT1 mRNA and protein levels decreased significantly in islets both *in vivo* and *in vitro*. MIN6 cells induced by palmitic acid exhibited increased apoptosis, and the accumulation of insulin and ATP in the supernatant decreased. Importantly, palmitic acid treatment combined with miR-204 silencing showed opposite changes. MiR-204 overexpression in MIN6 cells increased apoptosis and decreased insulin and ATP production and SIRT1 expression. SIRT1 overexpression reversed the damage to β-cells caused by miR-204. The BBR treatment effectively improved insulin synthesis, reduced miR-204 levels, and increased SIRT1 expression in islet tissue in diabetic mice. Overexpression of miR-204 reversed the protective effect of BBR on apoptosis and insulin secretion in MIN6 cells. Our study identifies a novel correlation between miR-204 and β-cell dysfunction in T2DM and shows that administration of BBR leads to remission of β-cell dysfunction by regulating the miR-204/SIRT1 pathway.

## Introduction

The progression of T2DM is a process in which pancreatic β-cells are continuously impaired or even fail in the context of insulin resistance (Abdul-Ghani et al., 2006). β-cell function damage marked by dysfunction of insulin synthesis and secretion and decreased β-cell numbers in islets are gradually aggravated or even play a decisive role in the development of T2DM (Ashcroft et al., 2017).

Sirtuin 1 (SIRT1), a nicotinamide adenine dinucleotide (NAD^+^)-dependent histone deacetylase, regulates multiple cellular processes by deacetylating related targeting proteins. It has been reported that SIRT1 activation has a beneficial effect on β-cell function and insulin sensitivity both in animal studies and clinical research (Kitada et al., 2019; Rehman et al., 2020; Zhou et al., 2018), suggesting that SIRT1 may be a promising new target for the treatment of T2DM. In terms of β-cell function, SIRT1 reduces the apoptosis of β-cells by inhibiting p53 acetylation. SIRT1 also increases insulin secretion by inhibiting UCP2 nuclear transcription (Huang et al., 2008). The relationship between SIRT1 and β-cell function has attracted our attention. However, as a key link in signal transduction, it is not clear how SIRT1 is regulated.

MicroRNAs (miRNAs) are noncoding small molecule single-stranded RNAs that act as important regulatory factors to mediate changes in downstream cell signaling pathways through the regulation of key proteins, thus affecting the various functions of cells (Mohr and Mott, 2015). Many studies have reported that miRNAs play an important role in pancreatic development and β-cell physiology. microRNAs for SIRT1 regulation was predicted through TargetScan and found that there were nine microRNAs with high scores. Among them, only miR-204 was enriched in islet and closely related to islet function, which was the upstream regulatory target of SIRT1 in corneal tissue. It has been reported that miR-204 was related to cell apoptosis and showed high expression in islet tissue of diabetic mice (Jiang et al., 2016; Li et al., 2019; Xu et al., 2013). Whether the expression of SIRT1 can be regulated by miR-204 to induce β-cell dysfunction has not been reported. It is also not clear whether the function of β-cells can be improved through inhibition of miR-204.

Berberine (BBR), an alkaloid extracted from *Coptis*, *Phellodendron* and *Berberis*, is a traditional Chinese medicine with remarkable anticancer, anti-inflammatory and antibacterial actions (Wang et al., 2017). In recent years, the excellent therapeutic effect of BBR on T2DM has been gradually studied. Many studies have attempted to demonstrate the potential mechanism by which BBR mitigates diabetes. Numerous studies have confirmed that BBR is a multi-target drug and its anti-diabetic activity is related to various mechanisms, including promoting insulin secretion, reducing insulin resistance, promoting glycolysis, inhibiting gluconeogenesis, inhibiting the inflammation response, and regulating intestinal microbial disturbance, etc (Xu et al., 2021). In improving insulin resistance, it is reported that BBR increase insulin sensitivity after 5 weeks administration in dietary obese rats and reduce HOMA-IR by 48% (Yin et al., 2008). BBR could also induce glycolysis by activating AMPK pathway and increasing GLUT1 and GLUT4 translocation (Lee et al., 2006; Liu et al., 2010). Moreover, Shan et al. found that BBR upregulated SIRT1 expression as a key regulator in adipose tissue, which contributions to the insulin sensing and anti-inflammatory effects of BBR (Shan et al., 2020). We pay more attention to the mechanism of BBR promoting insulin secretion. Zhou et al. (2009) studies show that BBR treatment significantly increase the insulin secretion, the number of β-cells, islet area, and ratio of pancreas to body weight in diabetic rats (Zhou et al., 2009). BBR could dose-dependently promote insulin secretion in HIT-T15 cells and mouse islets (Leng et al., 2004). Similarly, Wang et al. found that BBR exerted insulinotropic effect in isolated rat islets (Wang et al., 2008). Our previous studies and other articles have also found that BBR can suppress β-cell apoptosis and promote insulin release (Gao et al., 2011; Li et al., 2019). However, how BBR affects insulin secretion remains controversial. We also found that BBR treatment could significantly inhibit the expression of miR-204 in islet tissue in T2DM rats. In this study, we aimed to observe the role of miR-204 in β-cell dysfunction in T2DM and the correlation between miR-204 and the SIRT1 signaling pathway. We also explored whether BBR protects β-cells through this pathway, which will provide a new strategy for the mechanism of BBR.

## Materials and Methods

### Materials

BBR (purity quotient >99.8%) and streptozotocin (STZ) and palmitic acid (PA) were purchased from Sigma Chemicals Co., Ltd. (St. Louis, MO, United States). Glucose, total cholesterol (TC), triglyceride (TG) diagnostic test kits were purchased from Nanjing Jiancheng Bioengineering Institute (Nanjing, China). High-density lipoprotein cholesterol (HDL-C) and low-density lipoprotein cholesterol (LDL-C) ELISA kits were purchased from Tianjin Nine Tripods Medical and Bioengineering Co., Ltd (Tianjin, China). Insulin ELISA Kit was purchased from Merck Millipore Corporation (Darmstadt, Germany). Lipofectamine 2000 was purchased from Invitrogen (Carlsbad, CA, United States). The mouse or rabbit polyclonal antibodies were purchased from Abcam (Cambridge, MA, United States). Secondary antibodies were purchased from Proteintech (sa00001-1 and sa00001-2, USA). ATP Kit was purchased from Biyuntian Biotechnology (Beijing, China). BCA protein assay Kit was purchased from Thermo Fisher Scientific (USA). DMEM and Opti-MEM and Fetal bovine serum (FBS) were purchased from Gibco (Gibco, USA). MiR-204 mimic and inhibitor were synthesized and purchase from RioBio (Guangzhou, China). SIRT1 overexpression plasmid was conducted and purchase from GeneChem (Shanghai, China). All other reagents were purchased from Beijing Chemical Factory (Beijing, China).

### Animal Studies

The animal study designed in this paper was approved by the medical ethics committee of Jilin University. C57BL/6J male mice (weighing 22 ± 2 g) were purchased from Beijing HFK Bioscience Co., Ltd. (SCXK (Jing) 2014–0,004). The mice were housed individually in a temperature-controlled animal room with a constant 12 h light/dark cycle. The model of T2DM was established by high-fat feeding and low-dose intraperitoneal injection of STZ as described previously (Li et al., 2019; Zhang et al., 2008). After 1 week of adaptive feeding, they were randomly divided into two groups. The normal control group (Con, *n* = 10) was fed a low-fat diet (LF, *n* = 40) (10% kcal% fat, D12450B), and the type 2 diabetes model group (T2DM, *n* = 10) was fed a high-fat diet (HF, *n* = 40) (60% kcal% fat, D12492i, Research Diets, USA).

After 4 weeks, the model group was injected with a low dose of 100 mg/kg STZ (fasting for 12 h before injection). The control group was given the same amount of citrate buffer. One week later, the fasting blood glucose (FBG) of mice was detected by tail cutting. Mice with fasting blood glucose between 7.8 and 15 mmol/L were considered diabetic and included in the study. The mice were fed for 16 weeks, and then the glucose tolerance test (OGTT) and glucose-stimulated insulin secretion test (GSIS) were performed as described below. After that, mice were anesthetized with 100 mg/kg pentobarbital by intraperitoneal injection. Blood was collected from the orbital venous plexus, and serum was centrifuged at 3,500 × g for 15 min. Serum FBG, TC, TG and insulin were determined according to the manufacturer’s instructions. The pancreatic tissue was quickly removed from the mice, washed with cold normal saline, dried, half fixed with 10% formalin, and frozen in liquid nitrogen at −80°C until needed.

### Administration of Berberine to Type 2 Diabetic Mice

Thirty C57BL/6J mice purchased from Beijing from Beijing HFK Bioscience Co. Ltd. were randomly divided into three groups: the normal control group (Con, *n* = 10), the type 2 diabetes model group (T2DM, *n* = 10) and the BBR treatment group (BBR, *n* = 10). Based on our previous pharmacodynamic test, the optimal dose 160 mg/kg of BBR was chosen for this study (Wei et al., 2016). After modeling according to the above method, the BBR group was given 160 mg/kg BBR by gavage every day at 12 weeks, and the mice in the control group and model group were given 0.9% NaCl by gavage.

After 4 weeks of continuous drug treatment, OGTT and GSIS were measured 16 weeks before sacrifice and serum were obtained for biochemical analysis. The pancreas was stored at −80°C until needed.

### OGTT and GSIS

For the OGTT, mice were fasted for 12 h and then orally gavaged with glucose dissolved in water at 2 g/kg body weight. Ten microliters of blood were obtained from the tail tip, and the concentration of glucose was measured at 0, 30, 60, 90, and 120 min.

For GSIS, blood was collected from the orbital venous sinuses of mice for 0 min. Then, 40% (w/V) glucose was infused into the stomach according to 2 g/kg body weight. Blood samples were collected from the orbital venous sinuses at 15, 30 and 60 min. The content of insulin in serum was detected by a highly sensitive insulin ELISA kit, and the area under the curve was recorded and calculated.

### Histopathologic and Immunohistochemical Examination of the Pancreas in Mice

The pancreas was quickly removed from the mice, washed with normal saline, dried, and then fixed with 10% formalin. With increasing ethanol concentration, the tissue was dehydrated. The pancreatic tissue was sectioned with an HM340E microtome, and the thickness of the slices was 3 μM. After H&E staining, to detect insulin protein, paraffin sections were incubated with anti-insulin antibody overnight and then observed by the streptavidin biotin peroxidase complex method. the tissue and cell structure were imaged under an optical microscope (U-III Multipoint Sensor System; Nikon, Tokyo, Japan).

### Culture and Transfection of MIN6 Cells

Islet β-cell MIN6 cells, purchased from Shanghai Bogu Biotechnology Co., Ltd. were cultured in DMEM (25 mM glucose) supplemented with 10% FBS, 1% penicillin-streptomycin (PS) and 1% β-mercaptoethanol in an atmosphere of 5% CO_2_ at 37°C.

MIN6 cells were seeded in 24 well plates at a density of 2 × 10^4^ cells/well or in six well plates at a density of 10^5^ cells/well. DMEM (containing 10% FBS and 1% β-mercaptoethanol) without 1% penicillin-streptomycin was used to culture the cells. When the cells were cultured to 60–70% confluence, transfection began. First, the transfected mimic, plasmid and Lipofectamine 2000 were diluted in Opti-MEM for 5 min. Then, the diluted mimic and plasmid were added to the transfection reagent for double mixing for 20 min. Finally, the double mixed solution was added into the corresponding culture plate well, cultured in Opti-MEM without FBS for 4 h, and then replaced with normal DMEM (containing 25 mM glucose, 10% fetal bovine serum, 1% penicillin-streptomycin, 1% β-mercaptoethanol) to continue the culture until the end of the experiment.

### Injury Model of MIN6 Cells Induced by Palmitic Acid

MIN6 cells were seeded into 96 well plates. When the fusion degree of cells reached approximately 80%, the blank control group and the treatment group with different concentrations of palmitic acid (0.1, 0.2, 0.3, 0.4, 0.5, 0.6, 0.75, and 1.0 mM) were set up. After 24 h of cell treatment, the MTT method was used to detect the cell survival rate, and the optimal concentration of palmitic acid was selected to establish the MIN6 cell injury model.

### Selection of Protective Concentration of Berberine

MIN6 cells were seeded into 96 well plates. When the fusion degree of cells reached approximately 80%, the blank control group, palmitic acid treatment model group, and palmitic acid combined with different concentrations of BBR (2.5, 5, 10, 20 μM) treatment groups were set up. The survival rate of MIN6 cells was measured by the MTT method after 24 h of cell culture, and the optimal protective concentration of BBR in the injury model of palmitic acid was selected.

### Detection of Apoptosis by Flow Cytometry

Flow cytometric analysis was performed according to standard procedures. Briefly, after the treatment of MIN6 cells in each group, the cells were harvested, washed with cold PBS, and centrifuged at 1,500 rpm for 3 min. The cells were resuspended in annexin V binding buffer, and the cell density was controlled from 0.25 × 10^7^–1 × 10^7^ cells/ml. Then, 5 μl annexin V-FITC and 10 μl PI (propidium iodide) were added to the cell suspension and incubated in the dark (15 min), and 400 μl annexin V binding buffer was added. Finally, the samples were analyzed by flow cytometry.

### Western Blot

Western blotting experiments were performed as described previously (Yu et al., 2018). Briefly, total protein was extracted from pancreatic tissue or MIN6 cells and then quantified by a BCA protein assay kit. Each sample (80 μg) was separated from a 12% twelve alkyl sulfonate polyacrylamide gel, transferred to a polyvinylidene fluoride (PVDF) membrane (Bio-Rad), and sealed with 5% (w/V) BSA for 2 h. Then, membranes were probed with appropriate primary antibodies overnight at 4°C (Caspase-3, SIRT1, β-actin). After washing with TBST (10 mm Tris-HCl, 150 mM NaCl and 0.1% (V/V) Tween-20) 3 times for 5 min each time, the membranes were cultured with secondary antibodies for 2 h at room temperature. Enhanced chemiluminescence (ECL) was used to display protein bands, and images were generated with a GENE Imaging system (Tannon, China).

### Detection of Accumulated Insulin Secretion by MIN6 Cells

MIN6 cells were seeded into 24 well plates. When the fusion degree of cells reached approximately 80%, the cells were treated according to their groups. Supernatant and cell protein were collected, and then the insulin content in the supernatant was detected with an insulin ELISA kit and calibrated with the concentration of cell protein.

### Detection of ATP Content

For the cell samples, the cells were washed with cold PBS and 100 μl ATP detection lysate was added to each well. Then, the cells were centrifuged at 4°C and 12,000 g for 5 min and the supernatant was collected for cryopreservation. For the tissue samples, 100 μl lysate was added to every 10 mg tissue, homogenized on ice, and centrifuged under the same conditions, and the supernatant was collected for cryopreservation. The ATP concentration in the sample was detected by an ATP Kit according to the instructions.

### MiR-204 Quantification

Total RNA isolation from pancreatic tissue or MIN6 cells was performed as described previously (Kroh et al., 2010). Briefly, RNA was extracted from 2 × 10^4^/well cells or 100 mg frozen tissue using TRIzol reagent. To detect the miR-204 level, a special primer was obtained from GenePharma (Shanghai, China). Reverse transcription was performed with a kit (Transgen Biotech, China). RT-PCR was carried out at 95°C for 10 min, followed by 40 cycles of 95°C for 15 s and 60°C for 30 s on an ABI 7300 using Fast Start universal SYBR Green Master (Roche, USA). U6 was used as the internal reference of miR-204. The expression levels relative to the control were estimated by calculating 2−ΔΔCT (ΔCTsample − ΔCTcontrol).

### Statistical Analysis

Data are presented as the mean ± S.E.M. Between two groups, analyses were performed using Student’s t-test and then the F-test to compare the variances. Multiple group comparisons and analyses were performed based on a one-way ANOVA, followed by a post hoc Bonferroni test, as appropriate. A value of *p* < 0.05 was considered statistically significant and *p* < 0.01 was considered significantly different. Statistical analyses were carried out using GraphPad Prism 5.01 Software (USA).

## Results

### MiR-204 Is Closely Associated With β-cell Dysfunction in Type 2 Diabetes Mellitus

**FIGURE 1:**
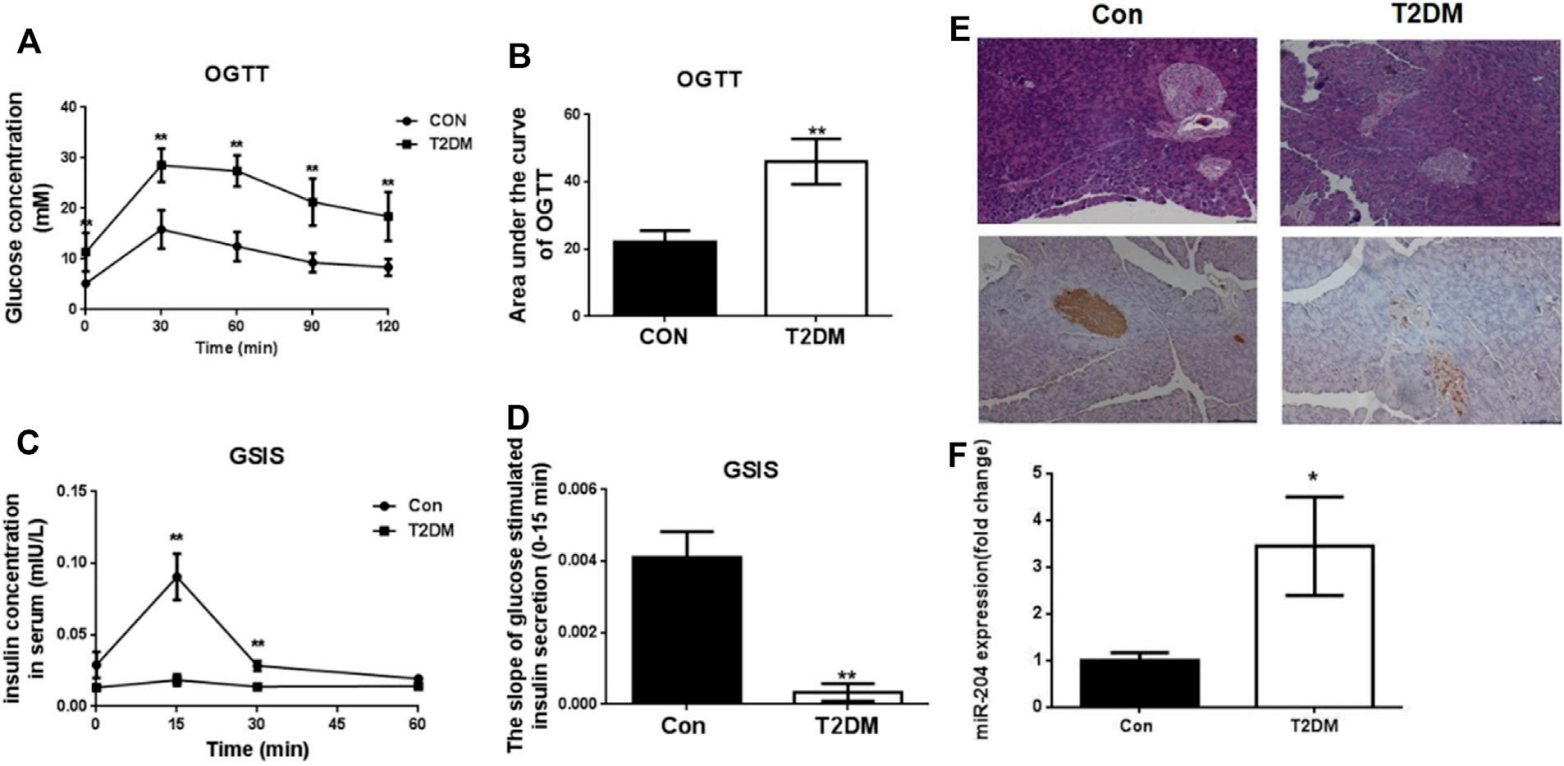
Changes of OGTT, GSIS, morphological of islets and miR-204 levels in type 2 diabetic mice at the end of 16 week. **(A, B)** After orally gavage with glucose, the curve of OGTT and the area under the curve were measured in the CON and T2DM groups. CON: control group; T2DM: type 2 diabetes mellitus group. OGTT: oral glucose tolerance test. **(C, D)** After stimulation of glucose, plasma insulin concentrations were measured in different phases during GSIS in CON and T2DM groups. GSIS: glucose stimulated insulin secretion (EI-EII) Histology were used to observe islet morphology in CON (left) and T2DM (right) mice (200×) (EIII-EIV) Pancreatic insulin level in CON (left) and T2DM (right) mice are measured using Immunohistochemistry (200×) **(F)** The RNA of islet tissue was extracted, and miR-204 levels was detected by RT-PCR in CON and T2DM groups. Data was expressed as mean ± SEM, *n* = 5, **p < 0.01 compared with CON group.

The changes in FBG, FINS, HDL-C, LDL-C, TG, TC and LDL-C in type 2 diabetic mice are shown in Table 1. The results showed that the FBG, LDL-C, TG and TC contents in T2DM mice were significantly higher while the FINS and HDL-C contents were obviously decreased, compared with the control group. The area under the glucose concentration curve (AUC) of the OGTT and GSIS (Figures 1A,B and Figures 1C,D) was observed in C57BL/6J mice fed for up to 16 weeks compared to the control group (Con). In the model group, glucose tolerance was impaired, and after glucose stimulation, the sensitivity of insulin secretion was significantly decreased. HE staining and insulin immunohistochemistry experiments of islet tissue sections were carried out to explore the morphological and functional changes of islets in diabetic mice. As shown in Figure 1E, compared with the Con group, the islet tissue area decreased, the shape of the islets tended to be irregular, and insulin secretion decreased significantly in the T2DM group, indicating that the number of islet cells and insulin secretion ability were obviously weakened in the T2DM group. To verify whether the above phenomenon is related to miR-204 in type 2 diabetic mice, the mRNA expression of miR-204 in islet tissue was detected by RT-PCR. The content of miR-204 in the islets of mice in the T2DM group was significantly higher than that in the control group (*p* < 0.01) (Figure 1F).

**TABLE 1:** Serum biochemical parameters of CON and T2DM C57BL/6J mice.

Parameter	Con	T2DM
FBG (mM)	3.16 ± 0.25	11.77 ± 1.71**
LDL-c (mM)	0.26 ± 0.02	0.53 ± 0.10*
TG (mM)	1.40 ± 0.04	2.23 ± 0.24*
TC (mM)	5.03 ± 0.23	7.04 ± 0.42**
FINS (mU/L)	13.01 ± 3.36	5.85 ± 0.20*
HDL-c (mM)	2.75 ± 0.15	2.20 ± 0.14*

Con: control group; T2DM: type 2 diabetic group. T2DM is induced by high-fat feeding and low-dose intraperitoneal injection of streptozotocin (STZ). FBG: fasting blood glucose; LDL-c: low-density lipoprotein cholesterol; TG: triglyceride; TC: total cholesterol. FINS: fasting insulin; HDL-c: high-density lipoprotein cholesterol. FBG, LDL-c, TG, TC, FINS, and HDL-c were determined at week 16. Data were expressed as the means ± SEM (*n* = 6). *p < 0.05, **p < 0.01 compared with Con group.

**FIGURE 2:**
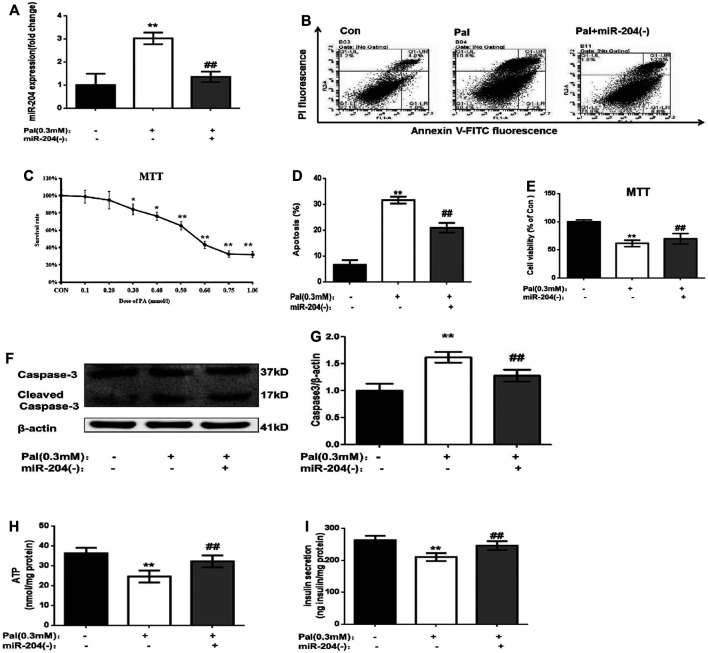
Palmitate (PA) induce the apoptosis in MIN6 cells and miR-204 silencing can mitigate this injury. After treated with PA for 24 h in normal cells **(A)** miR-204 expression was measured by RT-PCR. Con: control group, Pal:PA-treated MIN6 group, Pal + miR-204 (−):PA-treated + miR-204 (−) MIN6 group **(B–D)** The apoptosis was observed and analyzed by flow cytometry **(C)** The doses of PA-induced cytotoxicity in MIN6 cells were assessed by MTT assay. **(E)** The influence of PA-induced cytotoxicity and the mediation of miR-204 silencing were assessed by MTT assay. **(F, G)** The protein was extracted from cells and caspase3 were measured by Western Blot (H) ATP concentration in the supernatant of MIN6 cells was detected. **(I)** Basal insulin release and content were measured in MIN6. Data was expressed as means ± SEM (*n* = 6). *p < 0.05,**p < 0.01, compared with control group; ^##^ *p* < 0.01, compared with PA group.

To explore the role of miR-204 in islet injury, a MIN6 cell function damage model induced by palmitic acid (0.3 mM) was used (Figure 2C), and miR-204 (−) intervention was administered. Palmitic acid treatment increased the apoptosis of MIN6 cells (Figures 2B,D,E) and the protein expression level of Caspase-3 (Figures 2F,G) and decreased the basic accumulated insulin (Figure 2I) and ATP content in the supernatant (Figure 2H). The expression of miR-204 was increased 3.1-fold as much as that in the control group (Figure 2A). However, miR-204 silencing partially reversed the effect of PA. It is suggested that miR-204 may be a key regulatory factor in the injury of pancreatic β-cells induced by hyperlipidemia.

### MiR-204 Induced β-cell Dysfunction Through SIRT1

#### Changes of SIRT1 in Islets of Type 2 Diabetic Models *in vivo* and *in vitro*

SIRT1 is an NAD + -dependent histone deacetylase that plays an active role in prolonging cell life and inhibiting apoptosis. In recent years, SIRT1 has been found to positively regulate insulin secretion by islet β-cells, inhibit inflammation and improve insulin resistance (Chen et al., 2013; Deng et al., 2010.).

**FIGURE 3:**
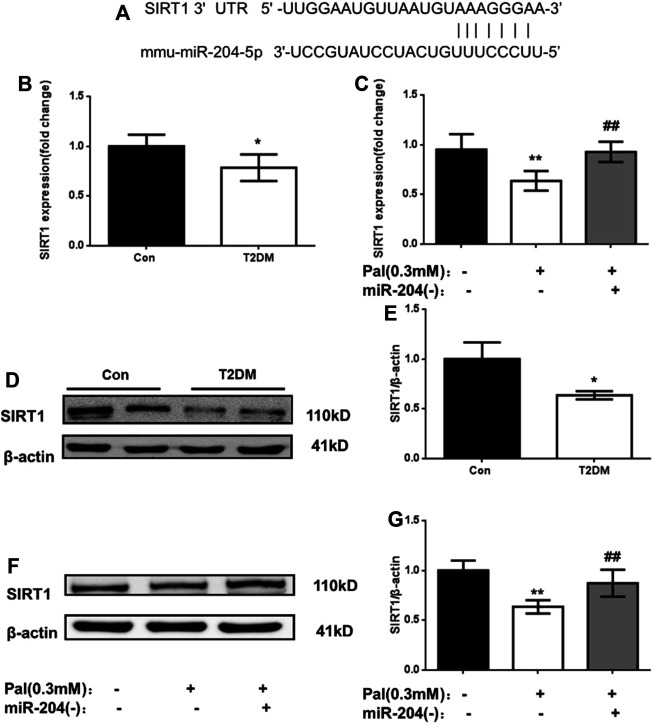
Changes of SIRT1 level in islets of type 2 diabetic models in vivo and in vitro. **(A)** The sequences of mmu-miR-204 and SIRT1 3′UTR complement each other by the prediction of targetscan. **(B)** RNA was exacted from islets tissue and SIRT1 was measured by RT-PCR and analyzed. Data was expressed as means ± SEM (n = 3). *p < 0.05, compared with CON group. **(C)** After treatment with PA and miR-204 (−), RNA was exacted from MIN6 cells and SIRT1 was measured by RT-PCR and analyzed. Data was expressed as means ± SEM (*n* = 3). **p < 0.01, compared with control group; ^##^ *p* < 0.01, compared with PA group **(D, E)** Protein was exacted from islets and SIRT1 was measured by Western blot and analyzed. Data was expressed as means ± SEM (*n* = 3). *p < 0.05, compared with CON group **(F, G)** The protein was extracted from cells and SIRT1 were measured and analyzed by Western Blot. Data was expressed as means ± SEM (*n* = 4). **p < 0.01, compared with control group; ##*p* < 0.01, compared with PA group.

MicroRNAs directly mediate the degradation or translation inhibition of target mRNA by combining with the complementary action sites of the 3′UTRs of target mRNA to widely regulate the expression of genes. Through a bioinformatics network, we predicted that the most likely upstream regulatory target of SIRT1 is miR-204 (Figure 3A). MiR-204 was enriched in islet tissue and was closely related to islet function. It has been proven that miR-204 can target and inhibit SIRT1 expression in the cornea. However, it is not known whether miR-204 can cause β-cell dysfunction by regulating SIRT1 expression in islet tissue.

To verify the relationship between miR-204 and SIRT1, firstly we detected the mRNA and protein levels of SIRT1 in the islet tissue of type 2 diabetic mice and in the MIN6 cell injury model induced by palmitic acid. As shown in Figure 3, the mRNA level of SIRT1 in the model group *in vitro* and *in vivo* was lower than that in the control group (*p* < 0.01), and the protein expression level of SIRT1 in the islet tissue of the T2DM group was also significantly decreased (*p* < 0.01). After miR-204 was silenced in the palmitic acid-induced MIN6 cell injury model, the SIRT1 mRNA and protein levels were significantly higher than those in the Pal group (*p* < 0.01). In combination with the corresponding miR-204 level changes in Figure 2, it can be inferred that miR-204 may has a regulatory effect on SIRT1 in the islet tissue.

#### MiR-204 Is an Upstream Regulator of SIRT1

**FIGURE 4:**
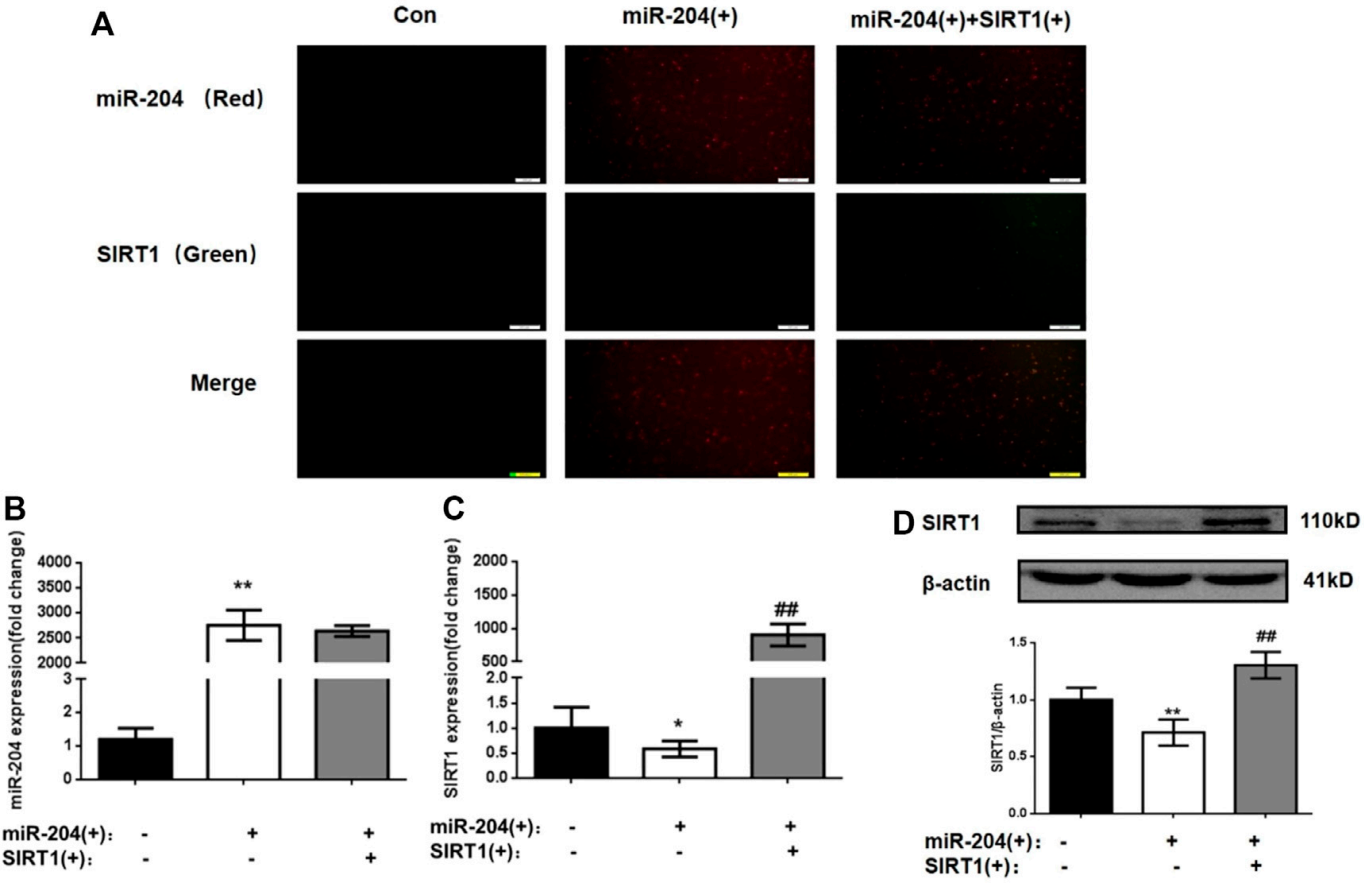
Changes of miR-204 level, SIRT1 mRNA and protein expression in MIN6 cells after plasmid intervention. **(A)**The transfection efficiency of MIN6 cells was verified. the transfected miR-204 mimic showed red fluorescence and the SIRT1 plasmid showed green fluorescence. **(B)** miR-204 was measured by RT-PCR after miR-204 and SIRT1 over-expression. Data was expressed as means ± SEM (*n* = 4). **p < 0.01, compared with control group. The mRNA **(C)** and protein level **(D)** of SIRT1 were measured after miR-204 and SIRT1 over-expression. Data was expressed as means ± SEM (*n* = 4). *p < 0.05, **p < 0.01, compared with control group; ##*p* < 0.01, compared with PA group.

In the following experiment, we used the miR-204 overexpression plasmid alone or combined with the SIRT1 overexpression plasmid to interfere with MIN6 cells to explore the relationship between miR-204 and SIRT1 as well as their effects on the apoptosis and function of MIN6 cells. To ensure the accuracy of subsequent experiments, the transfection efficiency of MIN6 cells was first verified. As shown in Figure 4A, the transfected miR-204 mimic showed red fluorescence and the SIRT1 plasmid showed green fluorescence. After 24 h of transfection, whether miR-204 mimic alone or miR-204 mimic co-transfected with SIRT1 plasmid, the transfection efficiency of MIN6 cells reached the requirement, and there was no effect on the survival rate of the cells.

As shown in Figures 4B–D, significant differences were not observed in the level of miR-204 between the miR-204 overexpression group and the miR-204 combined SIRT1 overexpression group. However, SIRT1 mRNA and protein expression decreased significantly when miR-204 was overexpressed alone. The results taken together indicated that miR-204 has an upstream regulatory effect on SIRT1, which can inhibit SIRT1 expression.

#### Effects of Overexpression of miR-204 and SIRT1 on Apoptosis and Insulin Secretion of MIN6 Cells

**FIGURE 5:**
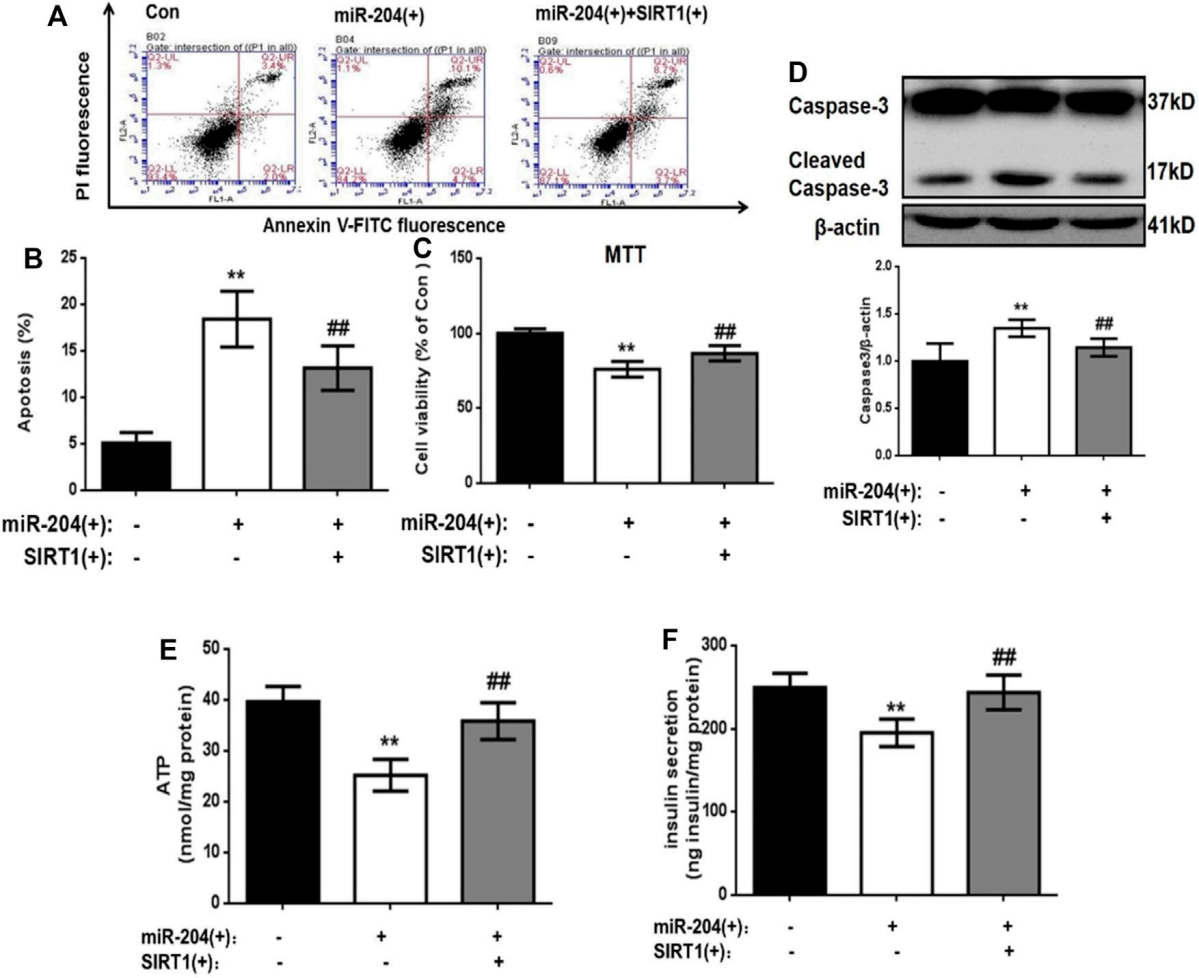
. Effects of overexpression of miR-204 and SIRT1 on apoptosis and insulin secretion of MIN6 cells. **(A, B)** After miR-204 overexpression or miR-204 combined with SIRT1 overexpression was given to MIN6 islet cells, the apoptosis was detected and analyzed by flow cytometry. Data was expressed as means ± SEM (*n* = 6). **p < 0.01, compared with control group; ^##^ *p* < 0.01, compared with miR-204 (+) group. **(C)** The influence of miR-204 overexpression and miR-204 combined with SIRT1 overexpression on the survival rate of MIN6 cells were assessed by MTT assay. Data was expressed as means ± SEM (*n* = 6). **p < 0.01, compared with control group; ^##^ *p* < 0.01, compared with miR-204 (+) group. **(D)** The protein was extracted from cells and caspase3 were measured by Western Blot. Data was expressed as means ± SEM (*n* = 3). **p < 0.01, compared with control group; ^##^ *p* < 0.01, compared with miR-204 (+) group. **(E)** ATP concentration in the supernatant of MIN6 cells was detected. Data was expressed as means ± SEM (*n* = 6). **p < 0.01, compared with control group; ^##^ *p* < 0.01, compared with miR-204 (+) group. **(F)** Basal insulin release and content were measured in MIN6. Data was expressed as means ± SEM (*n* = 6). **p < 0.01, compared with control group; ^##^ *p* < 0.01, compared with miR-204 (+) group.

The levels of insulin in the supernatant were measured 48 h after transfection. The results showed that miR-204 overexpression increased the apoptosis rate of MIN6 cells and increased the activity of Caspase-3. The simultaneous overexpression of miR-204 and SIRT1 partially reversed this trend (Figures 5A–D).

After overexpression of miR-204, the basic accumulated integral secretion of insulin was significantly lower than that of the control group (*p* < 0.01), while the insulin content in the simultaneous overexpression group of miR-204 and SIRT1 was significantly higher and close to the level of the control group (Figure 5F).

The process of insulin secretion in islet β-cells is regulated by many factors, in which the change in ATP content is very important. If the ATP content decreases, β-insulin secretion will be impaired. Therefore, ATP content can be used as one of the key indicators of insulin secretion energy metabolism. We detected the ATP content in the cells of each group 48 h after transfection. As shown in Figure 5E, in the miR-204 overexpression group, the ATP content in the cells was significantly lower than that in the Con group (*p* < 0.01), while this result was reversed in the miR-204 and SIRT1 overexpression group at the same time.

These results suggest that high expression of miR-204 can damage the function of pancreatic β-cells and the mechanism may be through the inhibition of SIRT1 mRNA expression.

### Mechanism by Which BBR Protects Against β-cell Functional Damage Induced by Hyperlipidemia Through the miR-204/SIRT1 Pathway

Our previous results showed that BBR could effectively improve the apoptosis of islet cells and decrease insulin secretion in the T2DM model group. To investigate its mechanism of action and whether the miR-204/SIRT1 pathway plays a role, we verified it at both the *in vivo* and cell levels.

**FIGURE 6:**
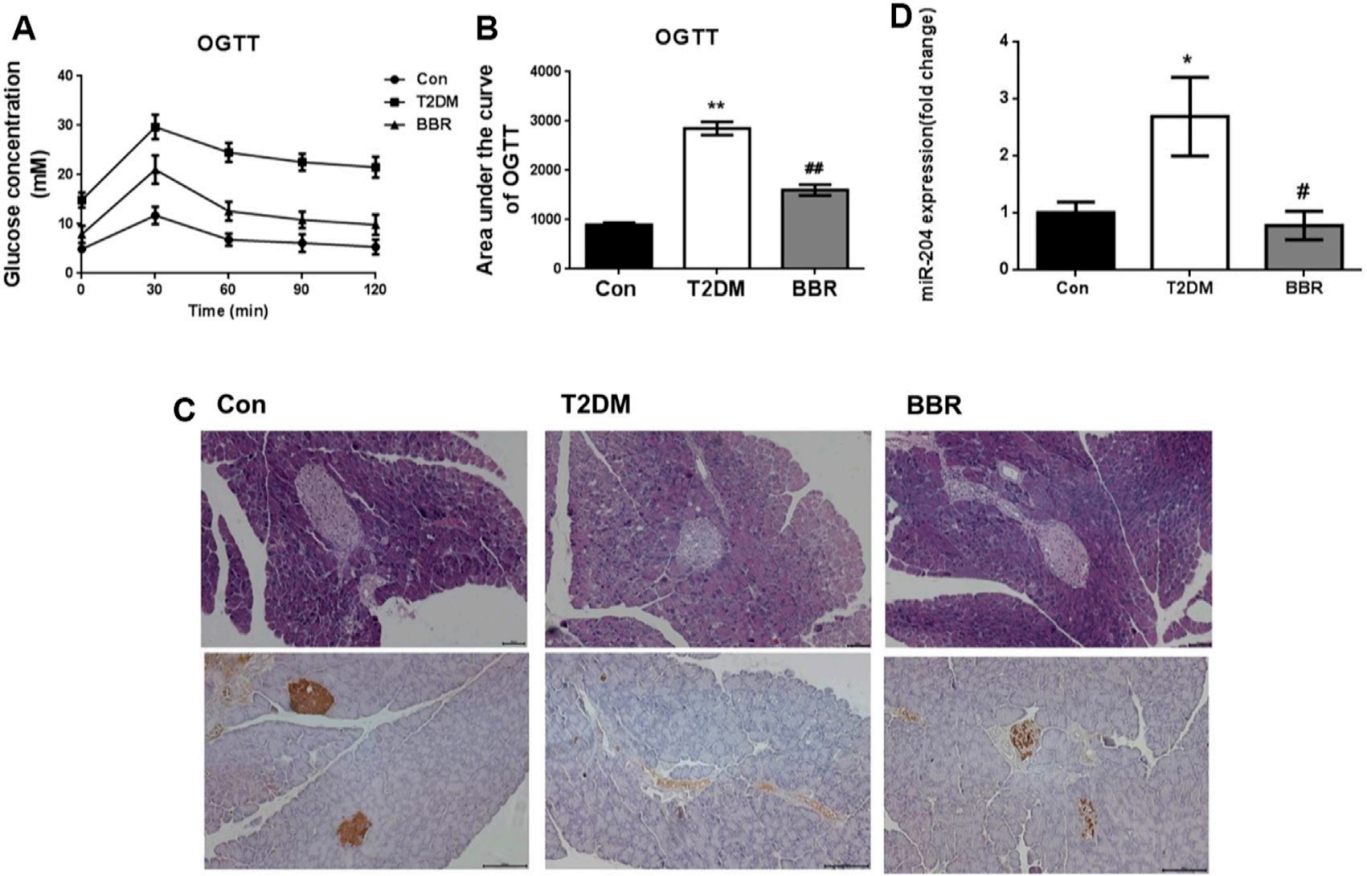
Effects of BBR on OGTT, islet morphology, insulin secretion and sera miR-204 levels in type 2 diabetic mice **(A, B)** After administration of BBR 160 mg/kg by gavage for 4 weeks, the curve of OGTT and the area under the curve were measured. CON: control group; T2DM: type 2 diabetes mellitus group. BBR: BBR treatment group. OGTT: oral glucose tolerance test. **(C)** Histology were used to observe islet morphology, and pancreatic insulin level are measured using Immunohistochemistry (200×) **(D)** The RNA of islet tissue was extracted, and miR-204 levels was detected by RT-PCR. Data was expressed as mean ± SEM, *n* = 5, *p < 0.05, **p < 0.01 compared with Con group; ^#^ *p* < 0.05, ^##^ *p* < 0.01 compared with T2DM group.

Treatment with BBR (160 mg/kg) for 4 weeks significantly reduced FBG levels and TG and TC contents, increased HDL-C content, and increased FINS levels in the model mice (Table 2). The OGTT curve trend and area under the curve of mice in the T2DM group were significantly higher than those in the Con group (*p* < 0.01), indicating insulin resistance in the model group. The condition of the BBR treatment group was improved (*p* < 0.01) (Figures 6A,B). The results of HE staining and insulin immunohistochemistry of mouse islets showed that BBR treatment could significantly improve the significantly reduced islet tissue area and insulin secretion in the T2DM group, which had a protective effect on islet cells and their functions (Figure 6C). RNA was extracted from the islet tissues, and miR-204 mRNA levels were detected in each group. The content of miR-204 in the islet tissues of the T2DM group was significantly higher than that of the Con group (*p* < 0.05), while the level of miR-204 in the BBR-treated group decreased significantly (*p* < 0.05), indicating that BBR could inhibit the expression of miR-204 (Figure 6D).

**TABLE 2:** Effect of BBR on the serum biochemical parameters of T2DM C57BL/6J mice.

Parameter	Con	T2DM	BBR
FBG (mM)	4.49 ± 0.51	15.21 ± 2.02 **	8.48 ± 0.51^##^
FINS (mU/L)	19.30 ± 2.00	11.37 ± 1.63 *	16.01 ± 1.08^#^
HDL-c (mM)	4.39 ± 0.44	2.74 ± 0.26 **	3.82 ± 0.12^##^
LDL-c (mM)	1.10 ± 0.24	2.12 ± 0.22 **	1.61 ± 0.18
TG (mM)	0.94 ± 0.05	1.71 ± 0.24 **	1.05 ± 0.08^#^
TC (mM)	3.31 ± 0.23	5.78 ± 0.31 **	4.67 ± 0.24^#^

**FIGURE 7:**
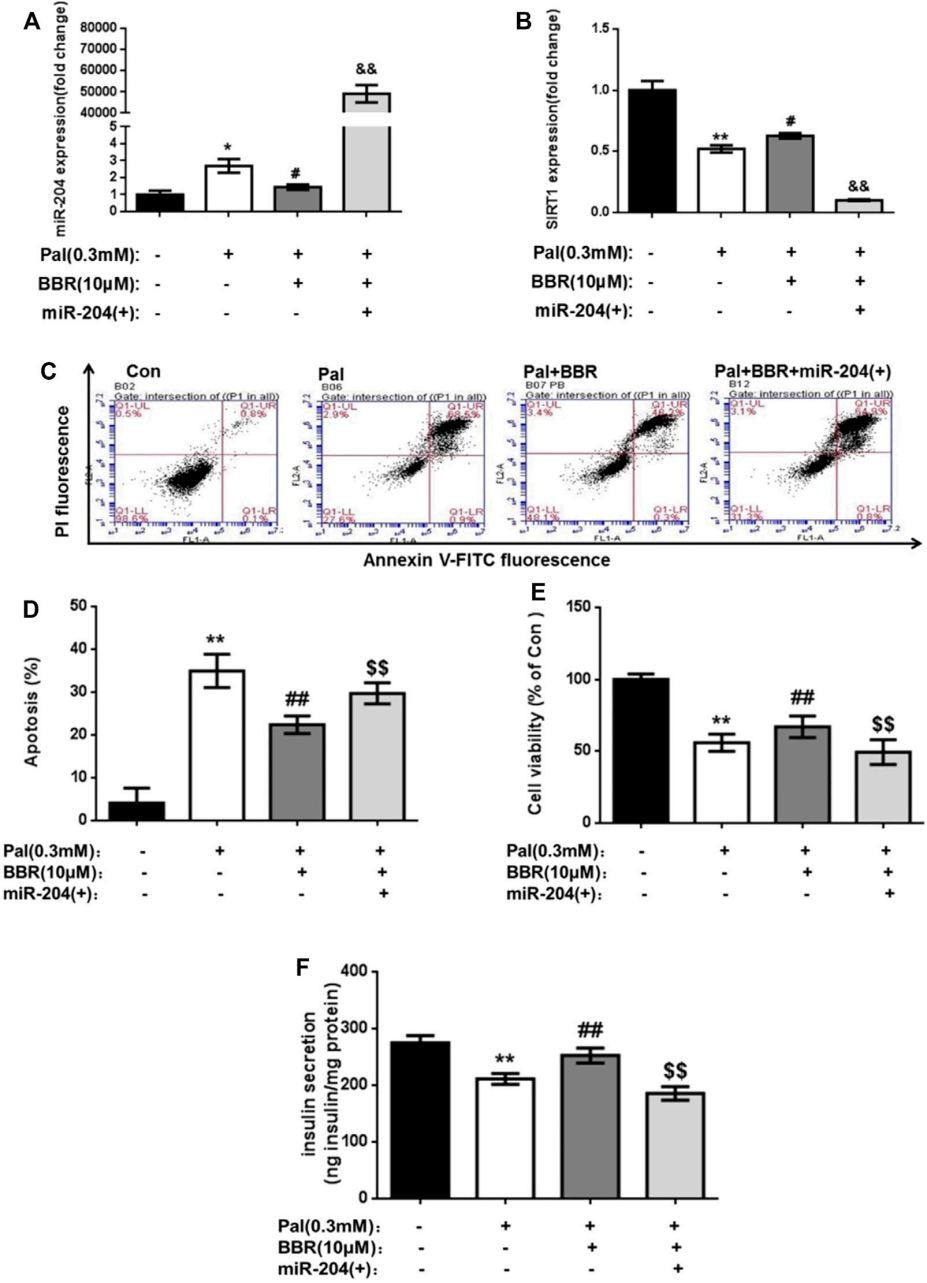
BBR may relieve the PA impairment on MIN6 cells via miR-204/SIRT1 pathway. After using BBR (10 μM) to treat PA-induced apoptotic and miR-204 overexpressed MIN6 cells for 24 h, the RNA of cells was extracted, and miR-204 expression **(A)** and SIRT1 expression **(B)** were measured by RT-PCR. **(C, D)** The apoptosis was observed and analyzed by flow cytometry **(E)** The influence on the survival rate of MIN6 cells were assessed by MTT assay. **(F)** Basal insulin release and content were measured in MIN6 cells. Data was expressed as means ± SEM (*n* = 6). *p < 0.05, **p < 0.01 compared with Con group; ^#^ *p* < 0.05, ^##^ *p* < 0.01 compared with Pal group; ^$$^ *p* < 0.01 compared with Pal + BBR group.

The apoptosis rate of MIN6 cells in the BBR group was significantly lower, and insulin secretion was increased compared with that in the Pal group, while the overexpression of miR-204 blocked the protective effect of BBR. This result suggested that the protective effect of BBR on Pal-induced apoptosis and insulin secretion of MIN6 cells might be realized through miR-204. RNA levels of miR-204 in each group were detected, and BBR treatment inhibited the increase in miR-204 levels in Pal-induced MIN6 cells and increased the expression of SIRT1, which was reversed when miR-204 was overexpressed (Figure 7). Therefore, we speculated that the protective effect of BBR on islet cells might be achieved by inhibiting the expression of miR-204 and indirectly promoting the expression of SIRT1.

Con: control group; T2DM: type 2 diabetic group. BBR: BBR treatment group. FBG: fasting blood glucose; LDL-c: low-density lipoprotein cholesterol; TG: triglyceride; TC: total cholesterol. FINS: fasting insulin; HDL-c: high-density lipoprotein cholesterol. After administration of 160 mg/kg BBR for 4 weeks, FBG, LDL-c, TG, TC, FINS, and HDL-c were determined. Data were expressed as the means ± SEM (*n* = 6). *p < 0.05, **p < 0.01 compared with the Con group; ^#^ *p* < 0.05, ^##^ *p* < 0.01 compared with the T2DM group.

## Discussion

Functional damage to β-cells plays a pivotal role in the development of T2DM, and high fat is one of the main risk factors for diabetes. In the process of T2DM, the long-term high level of free fatty acids (FFAs) in the body causes excessive insulin secretion and then leads to insulin resistance in peripheral tissues. However, a compensatory regulation series for β-cells resulted in endoplasmic reticulum stress, resulting in severe loss of insulin secretion function of β-cells and even apoptosis (Westermark et al., 1998; Butler et al., 2003; Maedler et al., 2003). Therefore, lipid toxicity-induced apoptosis of islet β-cells is currently considered one of the main pathogeneses and therapeutic target of T2DM, although its exact mechanism is not clear.

SIRT1 is an NAD + -dependent histone deacetylase that plays an active role in prolonging cell life and inhibiting apoptosis. In recent years, SIRT1 has been found to positively regulate insulin secretion by islet β-cells, inhibit inflammation and improve insulin resistance (Chen et al.; Deng et al.; Gaddam et al., 2020; Pinho et al., 2015). MicroRNAs directly mediate the degradation or translation inhibition of target mRNA by combining with the complementary action sites of the 3′UTRs of target mRNA to widely regulate the expression of genes. Through a bioinformatics network, miR-204 as the most likely upstream regulatory target of SIRT1 was predicted. MiR-204 was enriched in islet tissue and was closely related to islet function. It has been proven that miR-204 can target and inhibit SIRT1 expression in the cornea (Gao et al., 2015). However, it is not known whether miR-204 can cause β-cell dysfunction by regulating SIRT1 expression in islet tissue.

To explore the role of miR-204 in β-cell functional damage, C57BL/6J mice were given a high-fat diet combined with multiple low-dose intraperitoneal injections of streptozotocin (STZ) to establish a type 2 diabetic mouse model. Palmitic acid induces MIN6 cells to establish a functional damage model of islet β-cells *in vitro*. The results showed that the apoptosis rate of islet β-cells in the model group of type 2 diabetic mice and palmitic acid-induced cell model group obviously increased, while the level of insulin synthesis and secretion decreased significantly. Real-time PCR results showed that the miR-204 level in the model group increased significantly. After miR-204 silencing based on a cell-level palmitic acid-induced cell model, the phenomenon of increased apoptosis and decreased insulin secretion in the model group was improved, showing a protective effect on islet β-cells, indicating that miR-204 was a key regulator of islet cell dysfunction *in vivo* in type 2 diabetes or *in vitro* in the condition of simulated cell dysfunction induced by palmitoic acid.

It has been confirmed that miR-204 and SIRT1 combine and complement at the mRNA level in corneal tissue to inhibit their transcription (Gao et al., 2015). However, in islet tissue, which plays an important role in T2DM, it is not clear how the regulatory relationship between miR-204 and SIRT1 is; moreover, whether the increase in miR-204 in the diabetic state occurs through the influence of SIRT1 expression to participate in β-cell functional damage is also unclear. In the present study, we further speculated that miR-204 might be involved in the regulation of SIRT1 expression and affect the function of β-cells, thus affecting the pathogenesis of T2DM.

To verify our conjecture, we established a cell model of overexpression of miR-204 and combined overexpression of miR-204 and SIRT1. The results showed that the phenomenon of β-cell apoptosis and decreased insulin secretion in T2DM could be simulated *in vitro* by transfection of MIN6 cells with miR-204, that is, the functional damage of β-cells in islets. In the miR-204 overexpression model, the overexpression of SIRT1 effectively reduced the apoptosis of islet β-cells, increased the secretion of insulin, and protected the function of β-cells, suggesting that the functional damage of β-cells may be caused by miR-204 through regulating SIRT1 and then affecting downstream related proteins. The mechanism may be that miR-204, on the one hand, increases the apoptosis of islet beta cells by inhibiting the SIRT1/Caspase-3 pathway, and on the other hand, inhibit the secretion of insulin by inhibiting the SIRT1/UCP2/ATP insulin synthesis and secretion pathway, thus impairs the function of islet β-cells.

BBR is the main active component of Coptis chinensis. Many studies have confirmed that BBR has excellent antidiabetic effects (Lan et al., 2015), and BBR comes from natural plants, which has the advantages of few side effects and low price and has huge application prospects. Our previous studies have confirmed that BBR may prevent β-cell apoptosis and improve islet β-cell function in db/db mice and in palmitate-treated MIN6 cells (Li et al., 2019). However, the mechanism of BBR in diabetes, especially the functional mechanism of islet β-cells, is not clear. It has been reported that BBR can inhibit Th17 and Th1 cell differentiation in type 1 diabetic NOD mice by targeting MAPK, thus inhibiting T cell-mediated β-cell destruction and severe islet inflammation (Cui et al., 2009). BBR may have a protective effect on diabetes by increasing the expression of PARP-1 protein, increasing islet β-cell proliferation and antioxidant enzyme activity and reducing lipid peroxidation (Jiang et al., 2017; Zhou et al., 2009). In addition, it has been found that BBR can improve LPS-induced TLR4-independent inflammatory response-induced β-cell damage through the TLR4-independent JNK/NF-κB pathway (Wang, 2013). However, the mechanism of the protective effect of BBR on β-cell function is still unclear and needs more research.

To explore the protective mechanism of BBR on β-cell dysfunction, we observed the effect of BBR on β-cell apoptosis and insulin secretion function *in vivo* and at the cellular level. The results showed that BBR treatment can significantly increase the serum insulin level, effectively improve the phenomenon of atrophied islet area and decrease insulin synthesis in type 2 diabetic model mice. The content of miR-204 in islets of Langerhans increased, while the mRNA and protein levels of SIRT1 decreased. We further treated the PA-induced cell model with BBR, and the results were consistent with the integral animal experiment, confirming the protective effect of BBR on the functional impairment of islet cells. Based on palmitoic acid induction combined with BBR, we found that the therapeutic effect of BBR on islet cells in the model group disappeared after treatment with miR-204 overexpression in islet cells. This suggests that the protective effect of BBR on the function of diabetic islet cells may be achieved by inhibiting the miR-204/SIRT1 pathway.

**FIGURE 8:**
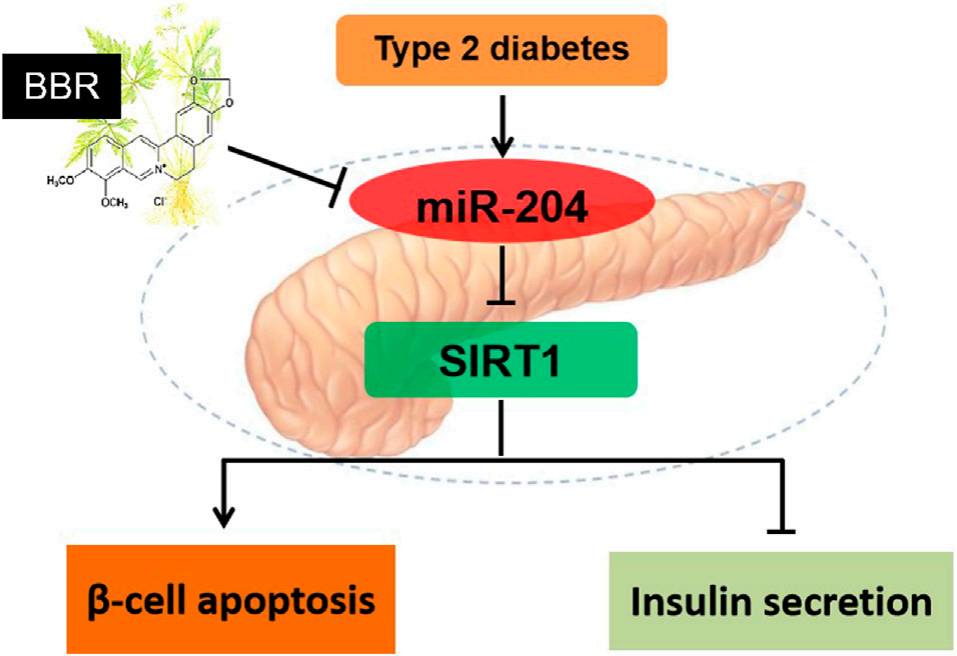
Schematic model of BBR in regulation of β-cell function. miR-204 inhibits SIRT1 to induce apoptosis of islet β-cells and decrease insulin synthesis and secretion, thereby leading to islet β-cell injury in T2DM. BBR alleviates β -cell dysfunction by regulating miR-204/SIRT1 pathway.

In conclusion, miR-204 inhibits SIRT1 to induce apoptosis of islet β-cells and decrease insulin synthesis and secretion, thereby leading to islet β-cell injury in T2DM. BBR alleviates β -cell dysfunction by regulating miR-204/SIRT1 pathway (Figure 8).

## Data Availability

The original contributions presented in the study are included in the article/Supplementary Material, further inquiries can be directed to the corresponding authors.
