# Supplementary material for: Berberine Potentiates Insulin Secretion and Prevents β-cell Dysfunction Through the miR-204/SIRT1 Signaling Pathway
Source: Front Pharmacol. 2021 Sep 22;12:720866. doi: 10.3389/fphar.2021.720866 (PMC8493072; doi:10.3389/fphar.2021.720866)
Supplement: Supplementary file 1 [file DataSheet2.docx]

**Figure legends**

**Figure 1. Changes of OGTT, GSIS, morphological of islets and miR-204 levels in type 2 diabetic mice at the end of 16 week.** (A-B) After orally gavage with glucose, the curve of OGTT and the area under the curve were measured in the CON and T2DM groups. CON: control group; T2DM: type 2 diabetes mellitus group. OGTT: oral glucose tolerance test. (C-D) After stimulation of glucose, plasma insulin concentrations were measured in different phases during GSIS in CON and T2DM groups. GSIS: glucose stimulated insulin secretion. (EI-EII) Histology were used to observe islet morphology in CON (left) and T2DM (right) mice (200×). (EIII-EIV) Pancreatic insulin level in CON (left) and T2DM (right) mice are measured using Immunohistochemistry (200×). (F) The RNA of islet tissue was extracted, and miR-204 levels was detected by RT-PCR in CON and T2DM groups. Data was expressed as mean ± SEM, n=5, **p<0.01 compared with CON group.

**Figure 2. Palmitate (PA) induce the apoptosis in MIN6 cells and miR-204 silencing can mitigate this injury.** After treated with PA for 24h in normal cells, (A) miR-204 expression was measured by RT-PCR. Con: control group, Pal：PA-treated MIN6 group, Pal+ miR-204(-)：PA-treated+miR-204(-) MIN6 group. (B, D) The apoptosis was observed and analyzed by flow cytometry. (C) The doses of PA-induced cytotoxicity in MIN6 cells were assessed by MTT assay. (E) The influence of PA-induced cytotoxicity and the mediation of miR-204 silencing were assessed by MTT assay. (F-G) The protein was extracted from cells and caspase3 were measured by Western Blot. (H) ATP concentration in the supernatant of MIN6 cells was detected. (I) Basal insulin release and content were measured in MIN6. Data was expressed as means ± SEM (n=6). *P<0.05,**P<0.01, compared with control group; ^##^P<0.01, compared with PA group.

**Figure 3. Changes of SIRT1 level in islets of type 2 diabetic models in vivo and in vitro.** (A) The sequences of mmu-miR-204 and SIRT1 3’UTR complement each other by the prediction of targetscan. (B) RNA was exacted from islets tissue and SIRT1 was measured by RT-PCR and analyzed. Data was expressed as means ± SEM (n=3). *P<0.05, compared with CON group. (C) After treatment with PA and miR-204(-), RNA was exacted from MIN6 cells and SIRT1 was measured by RT-PCR and analyzed. Data was expressed as means ± SEM (n=3). **P<0.01, compared with control group; ^##^P<0.01, compared with PA group. (D-E) Protein was exacted from islets and SIRT1 was measured by Western blot and analyzed. Data was expressed as means ± SEM (n=3). *P<0.05, compared with CON group. (F-G) The protein was extracted from cells and SIRT1 were measured and analyzed by Western Blot. Data was expressed as means ± SEM (n=4). **P<0.01, compared with control group; ##P<0.01, compared with PA group.

**Figure 4. Changes of miR-204 level, SIRT1 mRNA and protein expression in MIN6 cells after plasmid intervention.** (A)The transfection efficiency of MIN6 cells was verified. the transfected miR-204 mimic showed red fluorescence and the SIRT1 plasmid showed green fluorescence. (B) miR-204 was measured by RT-PCR after miR-204 and SIRT1 over-expression. Data was expressed as means ± SEM (n=4). **P<0.01, compared with control group. The mRNA (C) and protein level (D) of SIRT1 were measured after miR-204 and SIRT1 over-expression. Data was expressed as means ± SEM (n=4). *P<0.05, **P<0.01, compared with control group; ##P<0.01, compared with PA group.

**Figure 5. Effects of overexpression of miR-204 and SIRT1 on apoptosis and insulin secretion of MIN6 cells.**

(A-B) After miR-204 overexpression or miR-204 combined with SIRT1 overexpression was given to MIN6 islet cells, the apoptosis was detected and analyzed by flow cytometry. Data was expressed as means ± SEM (n=6). **P<0.01, compared with control group; ^##^P<0.01, compared with miR-204(+) group. (C) The influence of miR-204 overexpression and miR-204 combined with SIRT1 overexpression on the survival rate of MIN6 cells were assessed by MTT assay. Data was expressed as means ± SEM (n=6). **P<0.01, compared with control group; ^##^P<0.01, compared with miR-204(+) group. (D) The protein was extracted from cells and caspase3 were measured by Western Blot. Data was expressed as means ± SEM (n=3). **P<0.01, compared with control group; ^##^P<0.01, compared with miR-204(+) group. (E) ATP concentration in the supernatant of MIN6 cells was detected. Data was expressed as means ± SEM (n=6). **P<0.01, compared with control group; ^##^P<0.01, compared with miR-204(+) group. (F) Basal insulin release and content were measured in MIN6. Data was expressed as means ± SEM (n=6). **P<0.01, compared with control group; ^##^P<0.01, compared with miR-204(+) group.

**Figure 6. Effects of BBR on OGTT, islet morphology, insulin secretion and sera miR-204 levels in type 2 diabetic mice.** (A-B) After administration of BBR 160mg / kg by gavage for 4 weeks, the curve of OGTT and the area under the curve were measured. CON: control group; T2DM: type 2 diabetes mellitus group. BBR: BBR treatment group. OGTT: oral glucose tolerance test. (C) Histology were used to observe islet morphology, and pancreatic insulin level are measured using Immunohistochemistry (200×). (D) The RNA of islet tissue was extracted, and miR-204 levels was detected by RT-PCR. Data was expressed as mean±SEM, n=5, *P<0.05, **P<0.01 compared with Con group; ^#^P<0.05, ^##^P<0.01 compared with T2DM group.

**Figure 7. BBR may relieve the PA impairment on MIN6 cells via miR-204/SIRT1 pathway.** After using BBR (10μM) to treat PA-induced apoptotic and miR-204 overexpressed MIN6 cells for 24 h, the RNA of cells was extracted, and miR-204 expression (A) and SIRT1 expression (B) were measured by RT-PCR. (C-D) The apoptosis was observed and analyzed by flow cytometry. (E) The influence on the survival rate of MIN6 cells were assessed by MTT assay. (F) Basal insulin release and content were measured in MIN6 cells. Data was expressed as means ± SEM (n=6). *P<0.05, **P<0.01 compared with Con group; ^#^P<0.05, ^##^P<0.01 compared with Pal group; ^$$^P<0.01 compared with Pal+BBR group.

**Figure 8. Schematic model of BBR in regulation of β-cell function.** miR-204 inhibits SIRT1 to induce apoptosis of islet β-cells and decrease insulin synthesis and secretion, thereby leading to islet β-cell injury in T2DM. BBR alleviates β -cell dysfunction by regulating miR-204/SIRT1 pathway.


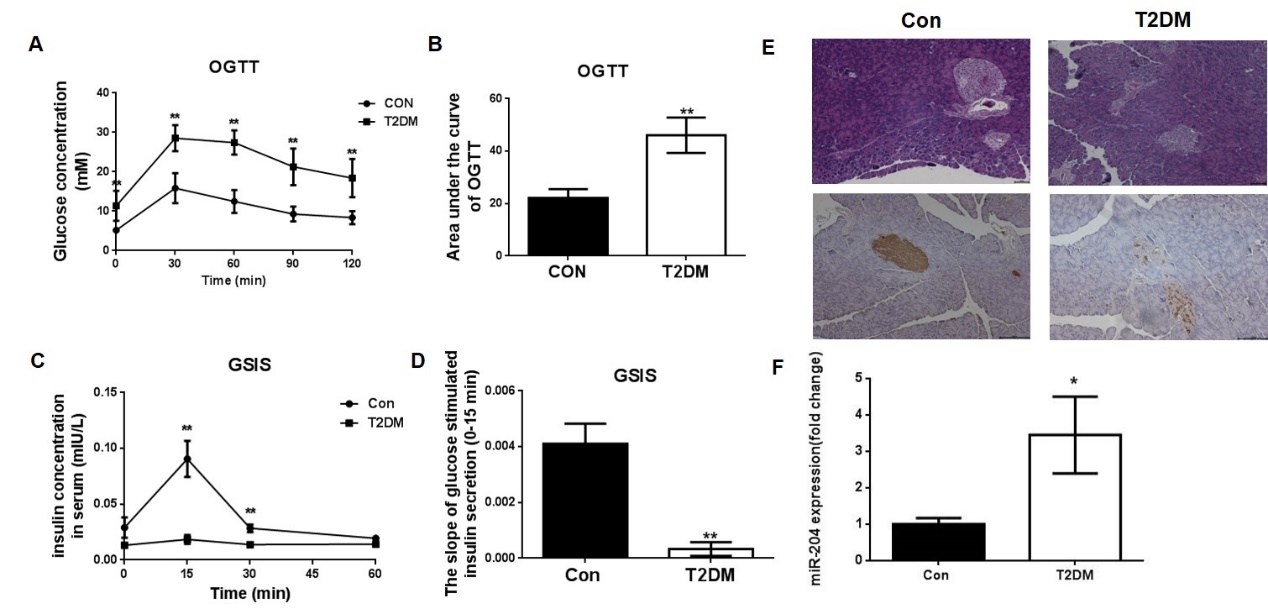


**Figure 1. Changes of OGTT, GSIS, morphological of islets and miR-204 levels in type 2 diabetic mice at the end of 16 week.**


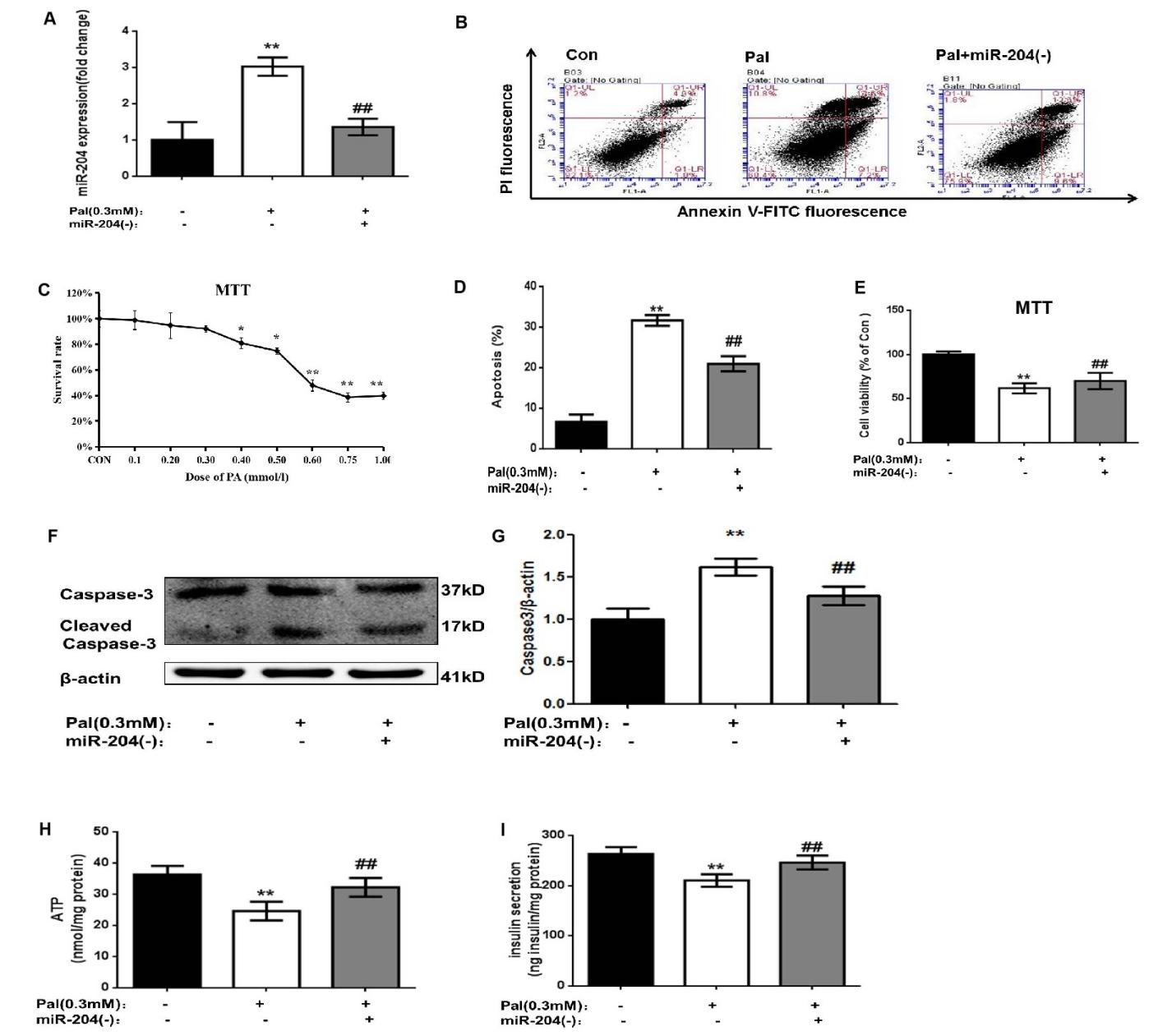


**Figure 2. Palmitate (PA) induce the apoptosis in MIN6 cells and miR-204 silencing can mitigate this injury.**


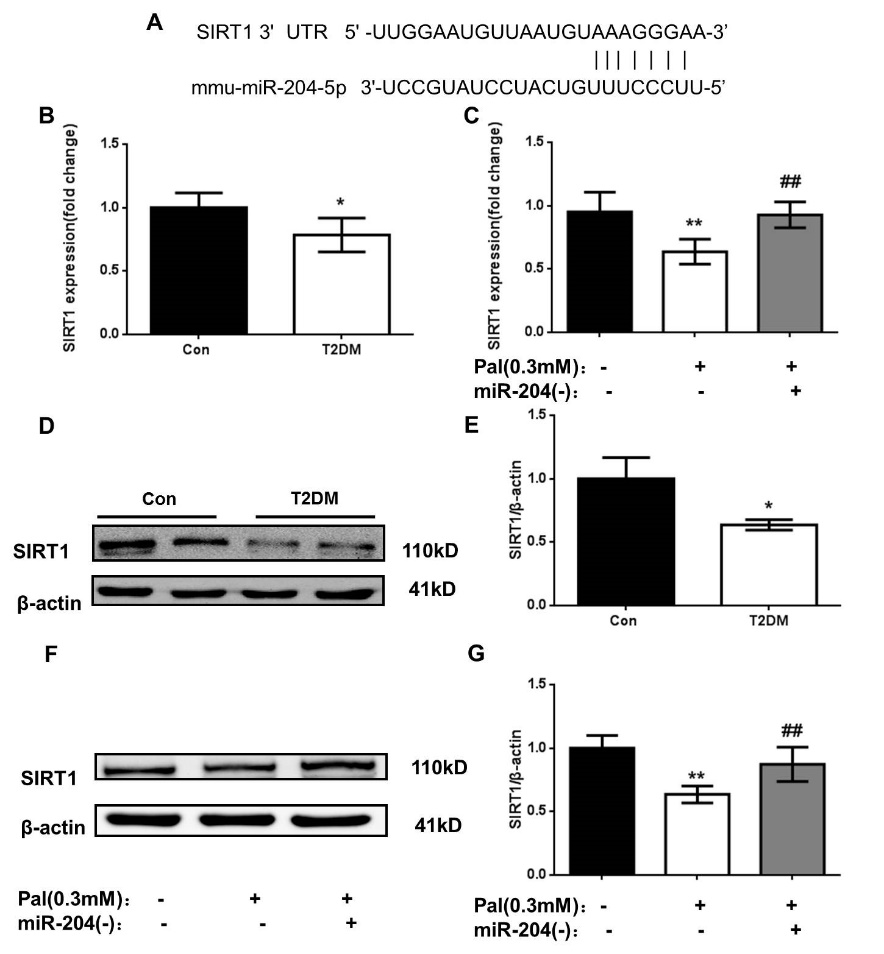


**Figure 3. Changes of SIRT1 level in islets of type 2 diabetic models in vivo and in vitro.**


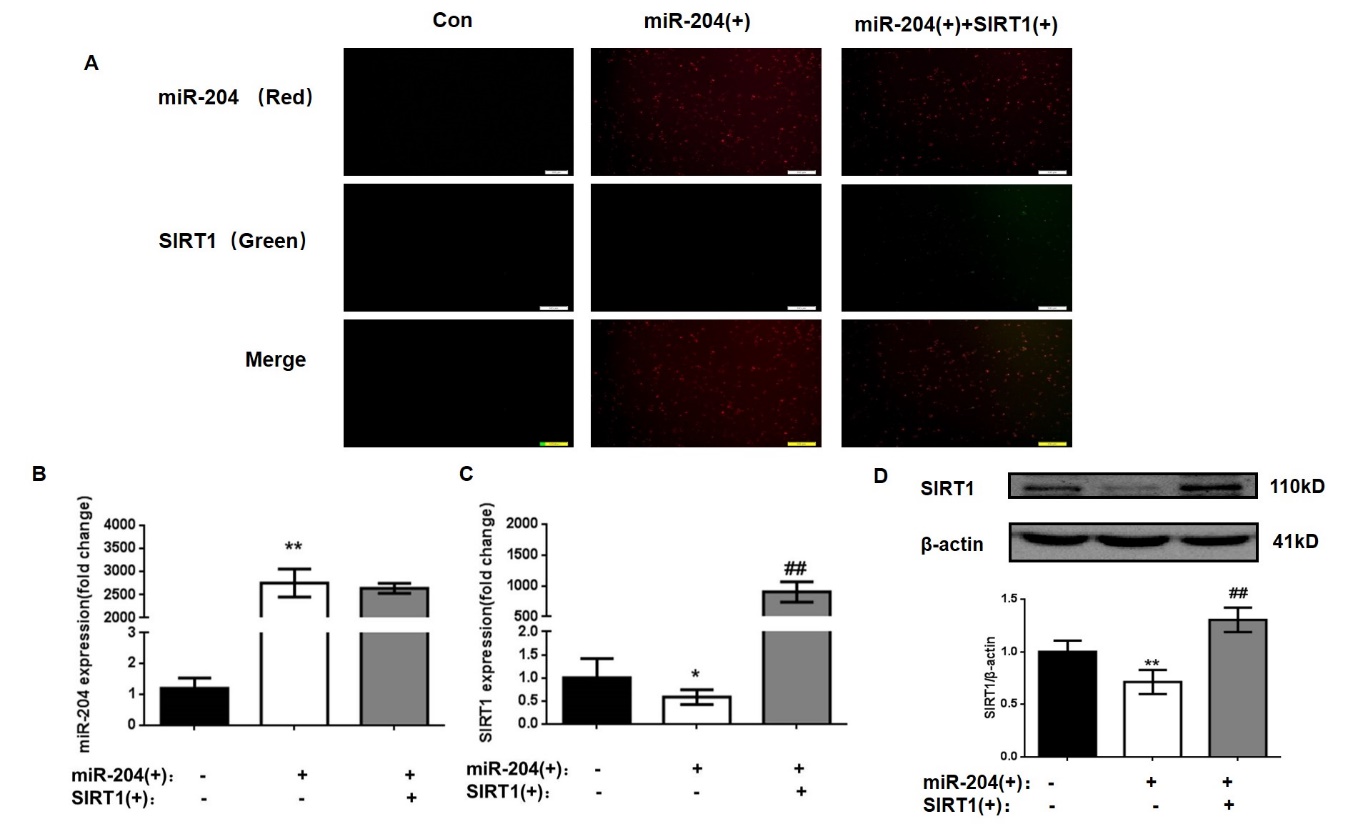


**Figure 4. Changes of miR-204 level, SIRT1 mRNA and protein expression in MIN6 cells after plasmid intervention.**


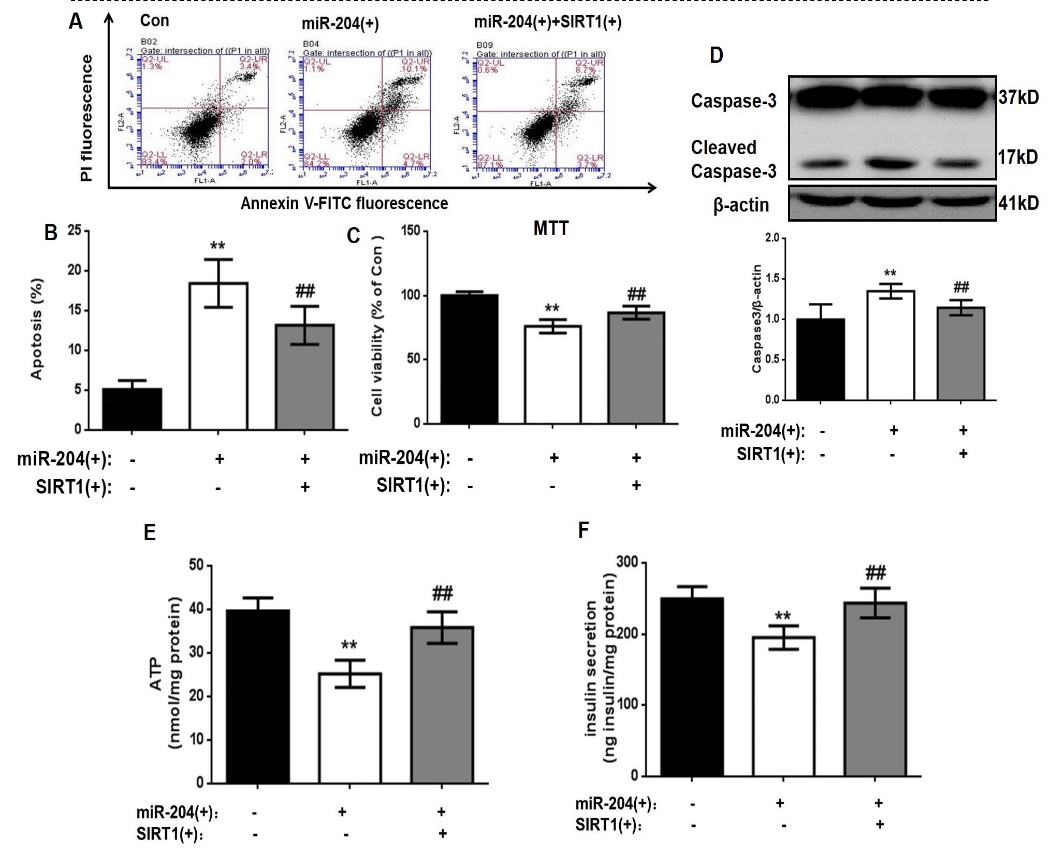


**Figure 5. Effects of overexpression of miR-204 and SIRT1 on apoptosis and insulin secretion of MIN6 cells.**


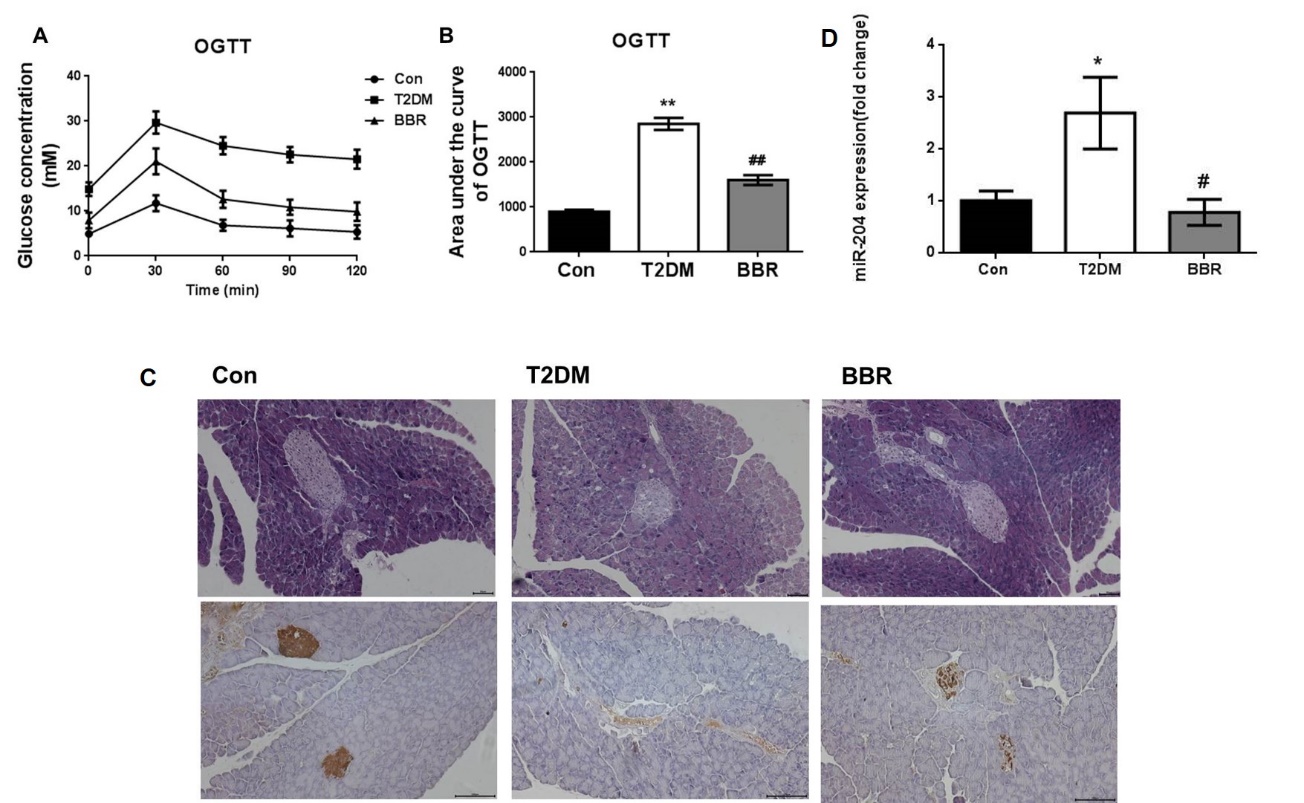


**Figure 6. Effects of BBR on OGTT, islet morphology, insulin secretion and sera miR-204 levels in type 2 diabetic mice.**


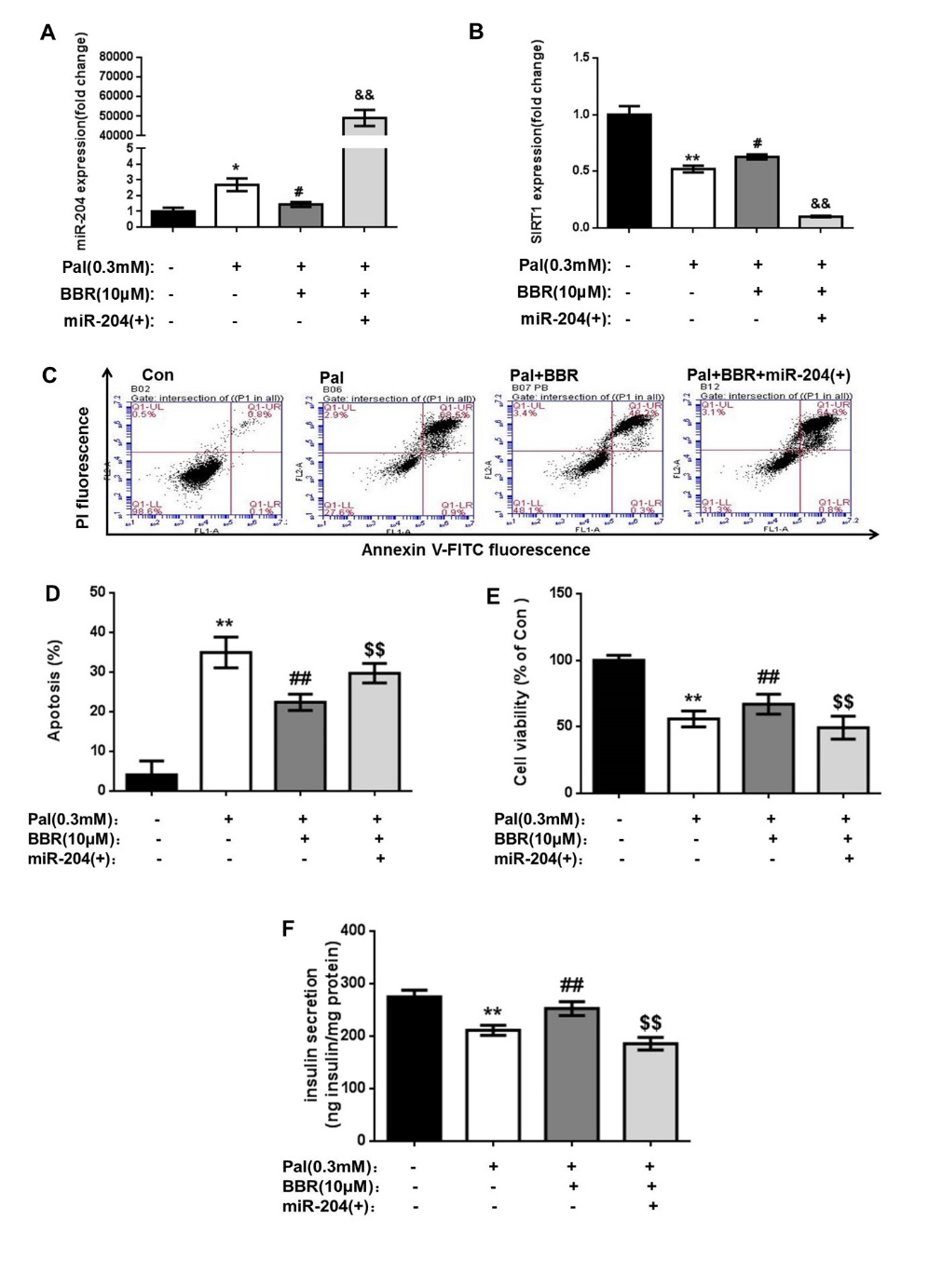


**Figure 7. BBR may relieve the PA impairment on MIN6 cells via miR-204/SIRT1 pathway.**


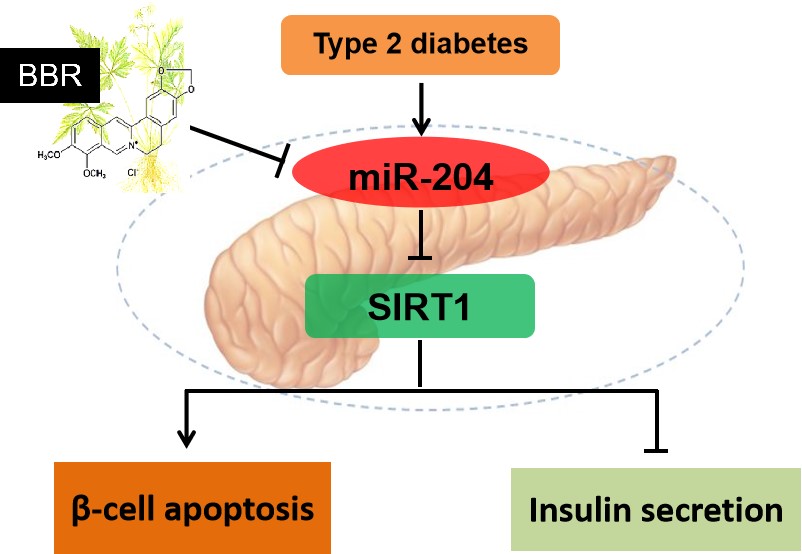


**Figure 8. Schematic model of BBR in regulation of β-cell function.**
