# Supplementary material for: Berberine Potentiates Insulin Secretion and Prevents β-cell Dysfunction Through the miR-204/SIRT1 Signaling Pathway
Source: Front Pharmacol. 2021 Sep 22;12:720866. doi: 10.3389/fphar.2021.720866 (PMC8493072; doi:10.3389/fphar.2021.720866)
Supplement: Supplementary file 2 [file DataSheet1.docx]

**CRediT Author Statement**

**Xiaoyan Lv:** Methodology, Writing- Reviewing and Editing; Funding acquisition.

**Yali Zhao:** Data curation, Writing- Original draft preparation.

**Xuehan Yang:** Methodology. Visualization.

**Hao Han:** Visualization, Investigation.

**Yue Ge:** Methodology.

**Meishuang Zhang:** Validation.

**Li Chen :** Supervision, Resources.

**Hansi Zhang:** Funding acquisition.

**Ming Zhang:** Conceptualization, Project administration.
